# Dual miRNA Targeting Restricts Host Range and Attenuates Neurovirulence of Flaviviruses

**DOI:** 10.1371/journal.ppat.1004852

**Published:** 2015-04-23

**Authors:** Konstantin A. Tsetsarkin, Guangping Liu, Heather Kenney, Jose Bustos-Arriaga, Christopher T. Hanson, Stephen S. Whitehead, Alexander G. Pletnev

**Affiliations:** Laboratory of Infectious Diseases, National Institute of Allergy and Infectious Diseases, National Institutes of Health, Bethesda, Maryland, United States of America; University of North Carolina at Chapel Hill, UNITED STATES

## Abstract

Mosquito-borne flaviviruses are among the most significant arboviral pathogens worldwide. Vaccinations and mosquito population control programs remain the most reliable means for flavivirus disease prevention, and live attenuated viruses remain one of the most attractive flavivirus vaccine platforms. Some live attenuated viruses are capable of infecting principle mosquito vectors, as demonstrated in the laboratory, which in combination with their intrinsic genetic instability could potentially lead to a vaccine virus reversion back to wild-type in nature, followed by introduction and dissemination of potentially dangerous viral strains into new geographic locations. To mitigate this risk we developed a microRNA-targeting approach that selectively restricts replication of flavivirus in the mosquito host. Introduction of sequences complementary to a mosquito-specific mir-184 and mir-275 miRNAs individually or in combination into the 3’NCR and/or ORF region resulted in selective restriction of dengue type 4 virus (DEN4) replication in mosquito cell lines and adult *Aedes* mosquitos. Moreover a combined targeting of DEN4 genome with mosquito-specific and vertebrate CNS-specific mir-124 miRNA can silence viral replication in two evolutionally distant biological systems: mosquitoes and mouse brains. Thus, this approach can reinforce the safety of newly developed or existing vaccines for use in humans and could provide an additional level of biosafety for laboratories using viruses with altered pathogenic or transmissibility characteristics.

## Author Summary

Despite advances in developing flavivirus live attenuated vaccine (LAV) candidates, a concern exists that they might not be safe in the environment due to their intrinsic genetic instability leading to potential reversion back to wild-type that could be associated with possible dissemination of these mutated viruses by mosquitoes. Here, we describe a miRNA targeting approach that can be adapted to support the design of environmentally-safe LAV restricted in their ability to infect and be transmitted by competent vectors, thereby limiting the possibility of subsequent viral evolution and unpredictable consequences. A combined co-targeting of flavivirus genome with mosquito- and vertebrate brain- specific miRNAs resulted in simultaneous restriction of dengue virus infection and replication in mosquitoes and in brains of newborn mice indicating that the miRNA-mediated approach for virus attenuation represents an alternative to non-specific strategies for the control of viral tissue tropism and pathogenesis in the vertebrate host and replicative efficacy in permissive vectors.

## Introduction

Mosquito-borne flaviviruses such as dengue, yellow fever, Japanese encephalitis and West Nile viruses (genus, *Flavivirus;* family, *Flaviviridae*) are among the most significant arboviral pathogens of humans and domestic animals in many regions of the world. Depending on the particular virus, clinical manifestations can vary from asymptomatic or self-limited flu-like illness to a life threatening jaundice, hemorrhagic fever and shock syndrome, or severe meningitis and encephalitis. Outbreaks and epidemics of flavivirus diseases typically coincide with warm rainy seasons, when the population of competent mosquito vectors reaches sufficient density for sustainable virus transmission between vertebrate hosts. Historically, mosquito control programs were considered as the most effective measure to prevent outbreaks and limit the spread of flavivirus diseases. However, reduction of funding allocated to mosquito control campaigns during the last decades, accompanied by an increase in human population density and global travel [1,2] resulted in an increase in the number of flavivirus-associated illnesses [1–3]. This emphasizes the need for development of effective vaccines or therapeutics as an alternative means for protection against flavivirus diseases.

A number of approaches are being pursued for development of effective vaccine candidates against flaviviruses, including RNA, DNA, inactivated, and subunit vaccines as well as vaccine candidates based on single-round replicated viral particles (reviewed in [4,5]). Although proven to be safe, they typically fail to provide long-lasting immunity after a single dose, and some may not be cost effective. This suggests a considerable advantage to vaccines based on live attenuated viruses that are relatively inexpensive to manufacture and provide a durable and potent immunity after a single dose of vaccination [6]. However, despite advances in developing live attenuated vaccine (LAV) candidates, a concern has been raised that they might not be safe in the environment due to their intrinsic genetic instability, potential reversion back to wild-type, and possible dissemination by mosquito vectors after feeding on vaccinees [7]. The possibility of mosquito transmission is further supported by the fact that many LAV candidates against diseases caused by mosquito-borne viruses can replicate in mosquito-derived cell lines and some are capable of infecting principle mosquito vectors in the laboratory [8–19]. For example, a vaccine strain of Venezuelan equine encephalitis virus (arbovirus, genus *Alphavirus*, family *Togaviridae*) was isolated outside its typical range from wild mosquitoes collected during a 1971 horse vaccination campaign in Louisiana [20], highlighting the risk of introduction and dissemination of potentially dangerous viral strains in new geographic locations.

Flaviviruses are enveloped single-stranded, positive-sense RNA viruses with genomes of approximately 11,000 nucleotide bases that contain 5’ and 3’-terminal non-coding regions (NCR) flanking a single open reading frame (ORF) encoding a polyprotein. During cap-dependent translation, the polyprotein is processed by viral and cellular proteases to yield three structural proteins (capsid [C], premembrane [prM], and envelope [E]) followed by at least seven nonstructural proteins (NS1, NS2A, NS2B, NS3, NS4A, NS4B, and NS5) [21]. In recent years, an approach based on targeting of viral genomes for number of cellular microRNAs (miRNAs) has been proven to be a simple and efficient method to restrict replication and pathogenesis of DNA and RNA viruses in a cell-, tissue- or species-specific manner [22–33]. Moreover, we demonstrated that insertion of targets for several brain-specific microRNAs (miRNAs) into the 3’ NCR and/or ORF of neurotropic chimeric tick-borne encephalitis virus/dengue type 4 virus (TBEV/DEN4) was sufficient to selectively inhibit viral replication in neurons and to constrain the development of lethal encephalitis in adult and newborn mice after intracerebral or intraperitoneal infection [34–36]. In this study using a similar miRNA-targeting approach, we sought to design a mosquito-borne flavivirus that would be selectively restricted for replication in its invertebrate host. As a model system to investigate the effect of miRNA targeting on flavivirus fitness in arthropod vector, we selected a dengue type 4 virus (DEN4) that efficiently replicates in widely distributed *Aedes (A*.*) aegypti* and *A*. *albopictus* mosquitoes. DEN4 is not neuroinvasive in vertebrate hosts and is currently being used as a genetic background for development of live attenuated vaccines against neurotropic and non-neurotropic flaviviruses [8–12]. However, the DEN4-based chimeric viruses, as well as the DEN4 parent, are able to replicate in the central nervous system (CNS) of neonatal mice infected intracerebrally, causing lethal encephalitis. Therefore, we also explored if combined targeting of the DEN4 genome with mosquito-specific and mouse brain-expressed miRNAs can simultaneously restrict DEN4 infection and replication in the mosquito host and attenuate virus neurovirulence in newborn mice.

## Results

### Insertion of a single copy of miRNA target into the 3’NCR attenuates DEN4 replication in mosquito cells and live mosquitoes

Recently, miRNA expression profiles have been identified for several mosquito species [37–39]. Based on this data, three mosquito-specific miRNAs (mir-184, mir-275 and mir-1) were selected for DEN4 genome targeting because they satisfy the following criteria: 1) they are highly expressed in different mosquito organs and mosquito-derived cell lines, and also remain abundant during flaviviruses infection [37]; 2) these miRNAs are evolutionarily conserved among insect species including mosquitoes, but they are different from their miRNA analogs in mammals.

**Fig 1:**
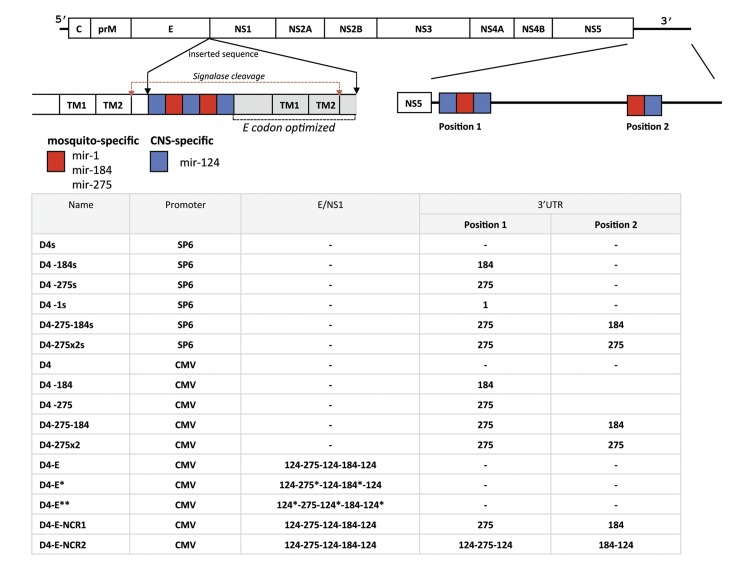
Schematic representation of viral genomes used in this study. Positions of miRNA targets for brain-expressed mir-124 and mosquito-specific mir-1, mir-184, or mir-275 in the ORF and 3’NCR of DEN4 genome are indicated by blue and red boxes, respectively. Gray area represents duplicated, codon optimized DEN4 E/NS1 sequence (nts from 2130 to 2451 of DEN4 genome) encoding 98 amino acids from the C-terminal end of the DEN4 E protein and 7 amino acids from the N-terminal end of the NS1 protein. TM1 and TM2 are transmembrane helixes in the C-terminal anchor region of protein E. Red arrows indicate signalase cleavage sites. Asterisk in the table indicates that miRNA target sequence has been altered using synonymous codons.

**Fig 2:**
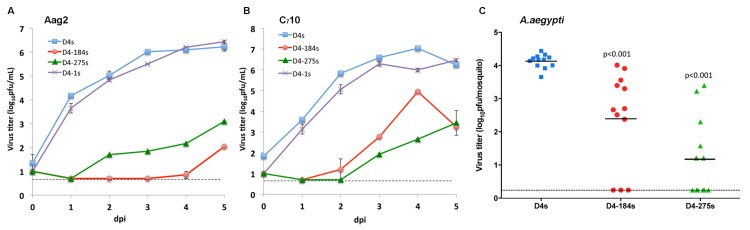
Effect of a single copy of miRNA target in the 3’NCR on DEN4 replication in mosquito cells and live mosquitoes. **(A** and **B)** Growth kinetics of D4s, D4-1s, D4-184s and D4-275s viruses in mosquito Aag2 (**A**) and C_7_10 (**B**) cells. Cells were infected at an MOI of 0.01. Each time point represents an average of two replicates ± standard deviation (shown as brackets). The dashed line indicates the limit of virus detection [0.7 log_10_ pfu/ml]. (**C)** A. aegypti *mosquitoes were infected* intrathoracically with 100 pfu of the indicated viruses and incubated for 7 days. Virus titer in each whole mosquito body suspension was determined in Vero cells. Each point represents the virus titer of an individual mosquito. Horizontal line represents the mean virus titer for all mosquitoes. The dashed line indicates the limit of virus detection [0.2 log_10_ pfu/mosquito]. P-values were calculated using unpaired one-tailed Student's t-test and adjusted using Bonferroni correction method to account for multiple comparisons.

To investigate if miRNA targeting of DEN4 genome results in selective restriction of DEN4 replication in mosquitoes, a single copy of mir-184, mir-275, or mir-1 target sequence was introduced into the genome of DEN4 strain 814669 [40] (abbreviated D4s) between nucleotides (nts) 10277 and 10278 (15 nts downstream of the TAA stop codon preceding the 3’NCR). This particular strain of DEN4 was chosen because it has been extensively characterized in the laboratory and is currently being used as a genetic background for construction of LAV candidates against flaviviruses [8–12]. DEN4 viruses carrying miRNA target sequences were generated by electroporation of *in vitro* transcribed full-length genomic RNA into Vero cells. Parental and each modified DEN4 virus (designated D4s, D4-184s, D4-275s, and D4-1s; Fig 1) were harvested on day 5 post-electroporation and amplified by one additional passage in Vero cells. Specific infectivity values of synthesized RNA and viral titers in Vero cells supernatants for D4-184s, D4-275s, and D4-1s were comparable to the unmodified D4s clone (S1 Table), indicating that insertion of target sequences into the 3’NCR did not result in substantial attenuation of DEN4 in Vero cells. The effect of miRNA targeting on the DEN4 replication in mosquito cell lines was analyzed by comparing growth kinetics of viruses in Aag2 and C_7_10 cells isolated from *A*. *aegypti* and *A*. *albopictus* mosquitoes, respectively. In both cell lines infected at multiplicity of infection (MOI) of 0.01, parental D4s and D4-1s viruses replicated efficiently with nearly identical kinetics reaching titers of ~6 log_10_ pfu/ml by 3 dpi (Fig 2A and 2B). In contrast, the replication of viruses D4-184s and D4-275s carrying a target for mir-184 or mir-275 was significantly impaired (p<0.001; 2-way ANOVA). In both cell lines, these viruses exhibited a 1000-fold or higher reduction in virus titer at 3 days post-infection (dpi) compared to the D4s virus, correlating with mir-184 and mir-275 expression levels in Aag2 and C_7_10 cell lines (S1 Fig) [37]. Based on these data, we selected D4-184s and D4-275s for further evaluation of their replication in adult *A*. *aegypti* mosquitoes.Female mosquitoes were infected intrathoracically with 100 pfu of D4s, D4-184s, or D4-275s virus. After 7 days of incubation, whole body homogenates of each mosquito were prepared individually, and titers were determined on Vero cell monolayers (Fig 2C). Insertion of either miRNA target sequence in the 3’NCR of DEN4 genome led to a significant restriction of virus replication in mosquito bodies (p< 0.001, one-tailed t-test), however, it was not sufficient to completely suppress the DEN4 infection.

### Combined targeting for mir-184 and mir-275 miRNAs in the 3’NCR greatly reduced the DEN4 replication in mosquito cells and Aedes mosquitoes

Previously, we demonstrated that multiple miRNA-targeting of flavivirus genome for brain-specific miRNAs in the 3’NCR results in increased efficiency of miRNA-mediated virus suppression in the CNS of mice compared to that observed for viruses containing only a single copy of the miRNA target [34,35]. Accordingly, we reasoned that a similar strategy should be applied to achieve a more reliable attenuation for DEN4 virus replication in cell culture and adult mosquitoes. Two additional constructs were developed based on D4-275s that contained additional target sequence for either mir-184 or mir-275 at nt position 212 of DEN4 3’NCR (Fig 1). Unfortunately, titers that these viruses achieved after recovery were not sufficient for their biological evaluation in mosquitoes (S1 Table).

**Fig 3:**
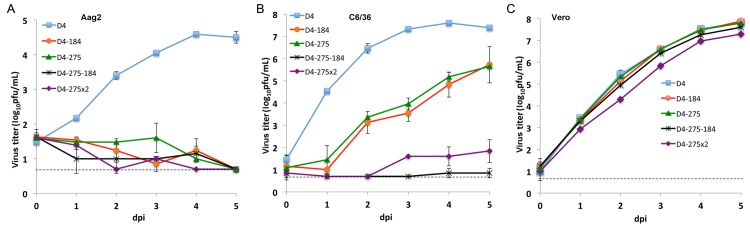
Effect of combined mir-184 and mir-275 co-targeting of DEN4 genome in the 3’NCR on virus replication in mosquito and Vero cells. Confluent monolayers of Aag2 (**A**), C6/36 (**B**) or Vero (**C**) cells were infected with D4, D4-184, D4-275, D4-275x2 and D4-275-184 viruses at an MOI of 0.01. Each time point represents an average of two replicates ± standard deviation (shown as brackets). The dashed line indicates the limit of virus detection [0.7 log_10_ pfu/ml].

To improve virus recovery, we modified the DEN4 cDNA clone in the following ways: substituted the SP6 promoter (for *in vitro* transcription) with the eukaryotic RNA polymerase II cytomegalovirus (CMV) promoter; introduced two intron sequences at nt positions 3922 and 8836 of the DEN4 genome to minimize plasmid DNA toxicity during propagation in *E*. *coli*; inserted the hepatitis delta virus ribozyme sequence at the 3’-end of the DEN4 virus genome to ensure correct release of authentic 3’-terminated RNA during transcription; and introduced the previously identified Vero cell-adaptive mutation (L_122_→F) into the NS4B protein gene to enhance replication in Vero cells [41–44]. The modified parental and mir-184- or mir-275-targeted DEN4 viruses (designated D4, D4-184, and D4-275; Fig 1) were re-generated by cDNA transfection into Vero cells (S2 Table). Two additional viruses (D4-275-184 and D4-275x2) were developed based on the D4-275 genome and contained a second target sequence for either mir-184 or mir-275 at nt position 212 of the 3’NCR (Fig 1). Viruses were biologically cloned by terminal dilution and amplified by one additional passage in Vero cells (S2 Table). Viruses were genetically stable and contained the miRNA target insertions as assessed by sequence analysis of viral genomes after 5 additional passages in Vero cells. All viruses containing miRNA targets were significantly attenuated compared to D4 virus for growth in *A*. *aegypti*-derived Aag2 cells (Fig 3A, p<0.001; 2-way ANOVA) and in *A*. *albopictus*-derived C6/36 cells, which replaced the previously used C_7_10 cells (Fig 3B, p<0.001; 2-way ANOVA). As anticipated, the combined expression of miRNA targets at two distant positions of the 3’NCR (D4-275-184 and D4-275x2) led to a more effective restriction of the virus replication in mosquito cells and resulted in at least a 3.5 or 6.0 log_10_ pfu/ml reduction in virus titer compared to D4 virus in Aag2 and C6/36 cells, respectively. In contrast, all viruses bearing one or two targets for mosquito-specific miRNAs replicated efficiently in Vero cells and the virus yield of each miRNA-targeted virus did not differ significantly from that of parental D4 virus (Fig 3C).

**Fig 4:**
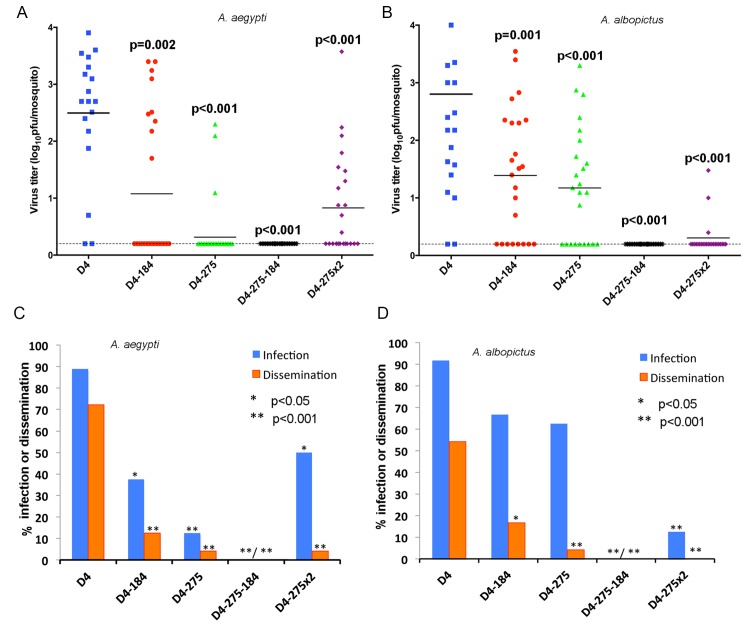
Effect of combined mir-184 and mir-275 co-targeting of DEN4 genome in the 3’NCR on virus fitness in A. aegypti *and* A. albopictus. A. aegypti *(* ***A*** *and* ***C*** *) and* A. albopictus *(* ***B*** *and* ***D*** *) were* orally infected with blood meals (BM) containing 7.1 to 7.4 log_10_ pfu/ml of recombinant D4 or D4-275-184 virus, respectively, and processed at 14 dpi. (**A** and **B**) Each point represents virus titer in individual mosquito (n = 18 for *A*. *aegypti* exposed to D4, and n = 24 mosquitoes for each other virus). Horizontal line represents an average titer for all mosquitoes. The dashed line indicates the limit of the assay detection [0.2 log_10_ pfu/mosquito]. P-values comparing mean titers of D4 and miRNA targeted viruses were calculated using unpaired one-tailed Student's t-test and adjusted using Bonferroni correction method to account for multiple comparisons. (**C** and **D**) Percentage of mosquitoes that became infected (blue) or developed a disseminated infection (orange) with each virus. Differences in infection and dissemination frequencies were compared between D4 and miRNA targeted viruses using one-tailed Fisher’s exact test. P-values were adjusted using Bonferroni correction method to account for multiple comparisons.

The maintenance of dengue viruses in the environment relies on the ability of the virus to infect, replicate, and develop a disseminated infection within two critical vectors: *A*. *aegypti* and *A*. *albopictus* mosquitoes. To investigate how targeting of the viral genome for mosquito-specific miRNA affects DEN4 fitness in female mosquitoes, *A*. *aegypti* and *A*. *albopictus* were fed with blood meals containing 7.1–7.4 log_10_ pfu/ml of recombinant DEN4 viruses. Viral titers in mosquito bodies, viral infectivity, and dissemination into heads of orally infected mosquitoes were assayed on day 14 after feeding. D4 virus infected 90% of both mosquito species by day 14, reaching a mean virus titer of ~2.5 log_10_ pfu/mosquito body, and disseminated to the heads of 71% of *A*. *aegypti* and 55% of *A*. *albopictus* (Fig 4). Introduction of a single copy of miRNA target for either mir-184 or mir-275 miRNA resulted in a significant reduction of the DEN4 titer in mosquito bodies (Fig 4A and 4B; p≤0.002 one-tailed Student's t-test) in both mosquito species as well as viral infectivity and ability of virus to develop a disseminated infection in *A*. *aegypti* (Fig 4C; p<0.05 one-tailed Fisher’s exact test). Infectivity of D4-184 and D4-275 viruses in *A*. *albopictus* were only slightly reduced as compared to D4 virus (Fig 4D; p = 0.144 and p = 0.0728), however both viruses were significantly attenuated in the ability to develop a disseminated infection (Fig 4D; p<0.05 one-tailed Fisher’s exact test). All D4-184 and D4-275 viruses recovered from *A*. *albopictus* and the majority of viruses recovered from infected *A*. *aegypti* contained deletions or point mutations in the miRNA target sequences (S2 Fig), indicating that targeting of the DEN4 genome by a single copy of miRNA target was not sufficient to prevent virus transmission by mosquitoes. In contrast, the D4-275-184 virus was unable to infect the midgut and thus failed to disseminate in both mosquito species indicating that a combined expression of mir-184 and mir-275 targets in D4-275-184 was sufficient to completely block DEN4 replication in the principal mosquito vectors (Fig 4C and 4D, p<0.001; Fisher’s exact test). These results are consistent with our previous observations made for viruses targeted by brain-specific miRNAs [34–36], suggesting that targeting of the viral genome in the same sites for two different miRNAs is a more efficient approach for flavivirus attenuation in the mosquitoes and the CNS of mice compared to a monotypic miRNA-targeting. Interestingly, a duplication of the target sequence for mir-275 miRNA alone resulted in a virus that was more infectious for *A*. *aegypti* (but not to *A*. *albopictus*) as compared to the D4-275-184 virus.

### Expression of mosquito-specific miRNA targets in the ORF region also results in a restriction of the mosquito vector range of the DEN4 virus

**Fig 5:**
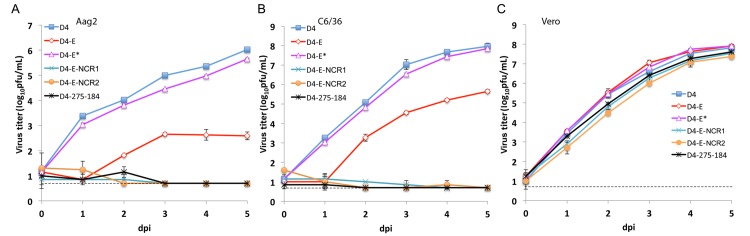
miRNA targeting of the DEN4 genome within the ORF and 3’NCR greatly attenuates virus replication in Aag2 and C6/36 cells but not in Vero cells. Confluent monolayers of Aag2 (**A**), C6/36 (**B**) and Vero (**C**) cells were infected in duplicate with either D4, D4-E, D4-E*, D4-275-184, D4-E-NCR1, and D4-E-NCR2 viruses at an MOI of 0.01. Each time point represents an average titer for two replicates ± standard deviation (shown as brackets). The dashed line indicates the limit of virus detection [0.7 log_10_ pfu/ml].

**Fig 6:**
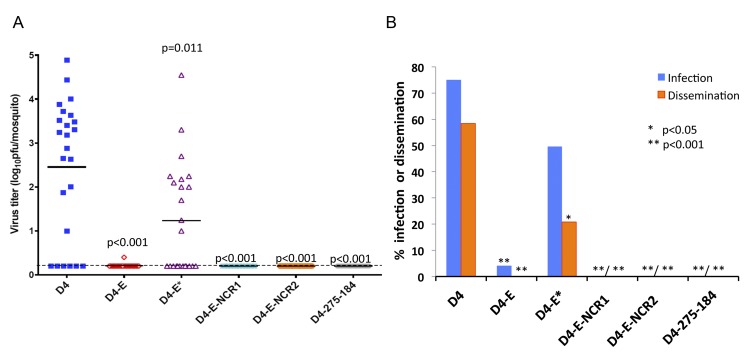
Effect of mir-184 and mir-275 co-targeting of DEN4 genome in the ORF and 3’NCR on virus fitness in A. aegypti *mosquitoes*. *A. aegypti* were presented with blood meals containing approximately 7 log_10_ pfu/mL of indicated recombinant viruses and processed after 14 days. (**A**) Each point represents virus titer in an individual mosquito (n = 24 mosquitoes per group). Horizontal line represents the mean virus titer for all mosquitoes in the group. The dashed line indicates the limit of assay detection [0.2 log_10_ pfu/mosquito]. P-values used to compare mean titers of D4 and miRNA targeted viruses were calculated using unpaired one-tailed Student's t-test and adjusted using Bonferroni correction method to account for multiple comparisons. (**B**) Percentage of mosquitoes that became infected (blue) or developed a disseminated infection (orange) with each virus. Differences in infection and dissemination frequencies were compared between D4 and miRNA targeted viruses using one-tailed Fisher’s exact test, and P-values were adjusted using Bonferroni correction method to account for multiple comparisons.

Previous studies of miRNA-regulated gene expression have demonstrated that the majority of mRNAs are post-transcriptionally regulated by targeting in the 3’NCR, and although regulation of mRNAs through miRNA-targeting in the coding region is less frequent, it is well documented (reviewed in [45,46]). Therefore, to explore if targeting of an ORF region of DEN4 by mosquito-specific miRNAs can result in specific viral attenuation in mosquitoes, targets for mosquito-expressed mir-184 and mir-275 as well as three targets for human neuron-specific mir-124 miRNA were introduced in the DEN4 genome between sequences encoding the two C-terminal stem-anchor domains of DEN4 E protein (D4-E virus; Fig 1). We designed these insertions of miRNA targets in this region using an approach that has been described previously [34,47]. As a control, we generated a D4-E* virus, in which mir-184 and mir-275 target sequences of D4-E were synonymously mutated in the third base position of each codon. The levels of replication for both control D4-E* virus and D4-E-mutant-bearing miRNA targets were similar in Vero cells indicating that the presence of miRNA targets or their scrambled sequences in the ORF of the viral genome does not affect viral fitness in cells that do not express the corresponding miRNAs (Fig 5C). Genome regions containing miRNA targets in D4-E or scrambled sequences in D4-E* virus remained stable for at least 5 subsequent passages in Vero cells as demonstrated by sequence analysis (S3 Fig). Replication of D4-E, but not D4-E*, was strongly attenuated in both Aag2 and C6/36 cells as compared to the D4 virus (Fig 5A and 5B, p<0.001; 2-way ANOVA), but the level of a residual replication of D4-E in either cell line was higher than that observed for the D4-275-184 virus carrying miRNA targets in the 3’NCR (Figs 3 and 5). Studies in orally infected *A*. *aegypti* mosquitoes demonstrated that infection rates of D4-E but not D4-E* were significantly decreased as compared to D4 (Fig 6B, p<0.001 and p = 0.151, respectively; one-tailed Fisher’s exact test). Infectivity of D4-E to *A*. *aegypti* was also significantly lower than that of D4-E* (1/24 versus 11/24; p = 0.0009; Fisher’s exact test) and none of the mosquitoes (0/24) had disseminated infection with D4-E virus compared to 20.1% (5/24) for D4-E* (p = 0.025; Fisher’s exact test). These results indicate that miRNA targeting of the D4-E genome within the ORF can specifically attenuate the virus for both mosquito-derived cells and mosquitoes due to the presence of authentic miRNA target sequences.

Even though a combined expression of mir-184 and mir-275 targets in the 3’NCR was sufficient to greatly restrict the D4-275-184 virus replication in mosquito cells and abolish infectivity in adult mosquitoes, an escape mutant lacking both authentic target sequences can theoretically emerge as a result of error prone flavivirus replication under miRNA-mediated selective pressure. To minimize the probability of such events, we generated a virus (D4-E-NCR1; Fig 1) expressing mir-184 and mir-275 target sequences in both the ORF and 3’NCR of DEN4 genome. As expected, the resulting virus was genetically stable and replicated well in Vero cells, but failed to initiate a productive infection in mosquito Aag-2 and C6/36 cells (Fig 5). Moreover, none of the *A*. *aegypti* mosquitoes that fed on a blood meal containing 6.8 log_10_ pfu/ml of D4-E-NCR1 became infected, or developed a disseminated infection into mosquito heads as tested at 14 dpi (Fig 6). These findings indicate that combined co-targeting of the DEN4 genome in the ORF and 3’NCR does not result in target interference during miRNA mediated flavivirus attenuation in mosquitos.

### Silencing of DEN4 replication in mice and mosquitoes by multiple miRNA co-targeting of the viral genome

**Fig 7:**
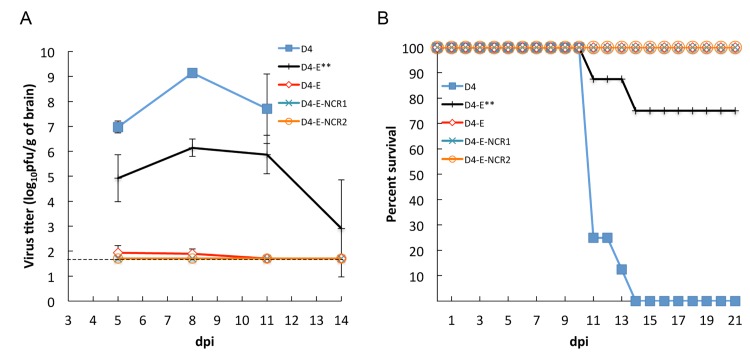
Effect of multiple mosquito- and brain-specific miRNA target insertions on DEN4 neurovirulence and replication in the brain of sucking mice. (**A**) Replication kinetics of D4, D4-E, D4-E**, D4-184, D4-E-NCR1, and D4-E-NCR2 viruses in the brains of mice. Three-day-old mice were inoculated IC with 3 log_10_ pfu of the indicated virus, and brains were collected from three mice in each group following the course of infection. Each time point represents an average titer for two replicates ± standard deviation (shown as brackets). The dashed line indicates the limit of virus detection [1.7 log_10_ pfu/ml]. Brain collection of mice infected with D4 and D4-184 was not performed at 14 dpi due to earlier paralysis of the animals. (**B**) Survival curves of sucking mice infected IC with 10^3^ pfu of recombinant DEN4 viruses (litter of eight 3-day-old mice per virus). Mice were monitored daily for morbidity for 21 days. We selected a 3 log_10_ pfu dose of virus inoculation since parental DEN4 strain 814669 has an IC LD_50_ of 407 pfu as estimated previously for this age of mice [48].

To explore if the miRNA targeting approach represents adequate means for simultaneous restriction of flavivirus replication in both the CNS of vertebrates and in mosquito vectors, we first evaluated neurovirulence of D4-E and D4-E-NCR1 viruses in a highly permissive animal model such as 3-day-old Swiss mice inoculated intracranially [34,36,42,44,48]. Both D4-E and D4-E-NCR1 viruses contained miRNA targets for mosquito-specific mir-184 and mir-275 and three copies of target sequences for vertebrate brain-specific mir-124 in the duplicated E/NS1 region (Fig 1). As a control virus for comparative assessment in the CNS of mice, we generated a D4-E** virus based on D4-E that contained synonymous mutations in the third base position of each codon of the CNS-specific mir-124 target sequences. The effect of mir-124 targeting in limiting neurotropism of flaviviruses has been extensively characterized in our laboratory previously [34–36]. Replication of D4-E and D4-E-NCR1 viruses in the mouse brain was strongly attenuated compared to that of D4 or D4-E** virus (Fig 7A, p<0.001 for each of control viruses; 2-way ANOVA). Moreover, none of the mice infected with D4-E or D4-E-NCR1 died, whereas the mortality rate for parental D4 was 100% (Fig 7B, p<0.001; log-rank test). This demonstrates that the miRNA targeting approach can be used for specific attenuation of flavivirus replication in more than one host or cell-type of interest. Interestingly, D4-E** virus with scrambled mir-124 target sequences in the ORF had a lower titer in the brain at each time point as compared with D4 virus (Fig 7A, p<0.001; 2-way ANOVA) and only 25% of mice infected with D4-E** virus died during a 21-days observation period (Fig 7B, p = 0.0014; log-rank test). This likely reflects that insertions of heterologous sequences (mir-275 and mir-184 targets) in the ORF can result in partial attenuation of DEN4 replication in the brain of mice.

Our previous studies [34,35] had demonstrated that increasing the number of miRNA targets in the 3’NCR of flavivirus genome significantly diminished virus replication in mouse brain and prevented virus escape from miRNA-mediated suppression. In order to minimize or reduce the emergence of DEN4 escape mutants during replication in the CNS, we developed an additional virus (D4-E-NCR2) containing a combination of brain and mosquito specific miRNA target sequences in the ORF and 3’NCR (Fig 1). As expected, the resulting D4-E-NCR2 virus efficiently replicated in Vero cells, but not in mosquito Aag2 or C6/36 cells (Fig 5; p<0.001; 2-way ANOVA). Moreover, no virus replication was detected in the brains of neonatal mice infected IC with D4-E-NCR2 (Fig 7A, p<0.001; 2-way ANOVA), and no death or neurological signs were observed (Fig 7B). In addition, the D4-E-NCR2 virus was unable to infect and generate disseminated infection in *A*. *aegypti* mosquitoes fed a blood meal containing 6.8 log_10_ pfu/ml of either virus (Fig 6B, p<0.001; Fisher’s exact test). We concluded that D4-E-NCR1 and D4-E-NCR2 viruses potentially could be used as a genetic background for development of chimeric live attenuated vaccine candidate against neurotropic flaviviruses, or a similar strategy could be applied for targeted attenuation of other flavivirus vaccine platforms such as yellow fever virus (YF) 17D or dengue viruses [13–19].

## Discussion

In addition to the low level of viremia observed following use of most flavivirus LAV, several additional factors that limit the potential for mosquito transmission have been identified, most of which are associated with non-specific or incidental attenuation of the vaccine strains. Prolonged serial passage of the Asibi strain of YF in cell cultures prepared from embryonated eggs led to generation of the YF 17D virus. Even though YF 17D is capable of infecting the *A*. *aegypti* midgut, the virus cannot be transmitted to vertebrate hosts due to its inability to escape the midgut barrier, which has been found to be associated with an accumulation of selective mutations in E, NS2A, and NS4B genes [49–52]. Similarly, a deletion of 30 nts (Δ30 mutation) in the 3’NCR of the DEN4 LAV platform not only led to virus attenuation in vertebrate hosts, but also resulted in a significant decrease in the dissemination rate in *Aedes* mosquitoes [53]. Both YF17D and DEN4Δ 30 viruses were subsequently used as genetic platforms for development of LAV candidates against dengue, West Nile, Saint Louis encephalitis, Japanese encephalitis, and tick-borne encephalitis viruses [8–19,40,43,44]. Analysis of the resulting vaccine viruses in mosquitoes demonstrated that genome chimerization also leads to an additional decrease in transmission potential of chimeric viruses constructed based on either 17D [13–19] or DEN4Δ 30 [8–12] genetic backgrounds.

In this study, using mosquito-borne DEN4 flavivirus as a model virus, we demonstrated the effectiveness of the miRNA targeting approach for the simultaneous attenuation of a virus in two evolutionally distant host organisms: mice and mosquitoes. Since miRNA-mediated control of viral tropism in mammals has been achieved previously for both RNA and DNA viruses, including flaviviruses [23–25,34–36,54], as well as selective species-specific attenuation in mammals [30], then silencing of virus replication or complete blockade of virus infectivity in the insect vector alone or in both vertebrate and invertebrate hosts represents the unique focus of this study. We suggest that this approach can be adapted to support the development of environmentally safe, live attenuated virus vaccines restricted in their ability to be introduced into nature during feeding of competent mosquito vectors on viremic vaccinees, thereby limiting the possibility for a subsequent evolution of LAV-derived viruses which can result in unpredictable consequences [7]. The miRNA-mediated silencing of virus replication in mice and mosquitoes demonstrated in our studies can reinforce the safety of newly developed and existing vaccines for use in humans.

The precise mechanism involved in miRNA-mediated attenuation of viruses in mosquitoes and their cell lines is unknown and requires further investigation. A variety of factors might play a role and contribute to the effectiveness of cellular miRNA-mediated RNA transcriptional repression and can depend on the level of miRNA expression, the number of miRNA targets and their locations in mRNA or viral RNA genomes, and their accessibility and interactions with the RNA-induced silencing complex (RISC) [23,45,46,55,56].

As demonstrated in our studies, increasing the copy number of miRNA targets was generally associated with more efficient DEN4 attenuation in mosquito cells and in *A*. *aegypti* and *A*. *albopictus* mosquitoes, presumably due to a greater number of RISCs becoming engaged in interactions with the DEN4 RNA genome. Among three selected targets for mosquito-specific miRNAs that were inserted into the 3’NCR of DEN4 genome, the presence of a target for the highly expressed miRNAs in mosquito cells Aag2 or C_7_10 (mir-184 and mir-275) reduced DEN4 replication to a greater extent than the inclusion of a target for the less expressed mir-1 miRNA (Figs 2 and S1) [37]. This indicates that inhibition of DEN4 replication occurs by a miRNA-mediated mechanism and is not a result of unspecific attenuation of viral replication due to insertion of heterologous sequences into the 3’NCR. This also suggests that miRNAs with the highest mosquito cell abundance should be used for effective viral genome targeting.

As described previously [57], insects have two functionally distinct RNase III enzymes (Dicers): Dicer-1 is primarily involved in biogenesis of cellular miRNAs, whereas Dicer-2 participates in the RNA interference (RNAi) response through production of small interfering RNAs from extended double stranded RNAs. It has been demonstrated that unlike Aag2, *A*. *albopictus* derived C6/36 cells exhibited impaired RNAi response due to aberrant Dicer-2 gene expression [58,59]. Since replication of miRNA-targeted D4-184 and D4-275 viruses in both Aag2 and C6/36 cell lines was attenuated compared to parental D4 virus, it can be assumed that inhibition of viral replication occurs primarily through a Dicer-1-dependent miRNA-mediated mechanism, but not through a Dicer-2-mediated RNAi mode (Fig 3).

Placement of miRNA targets in the 3’NCR has an overall stronger suppressive effect on the DEN4 replication in mosquito cells than target placement in the ORF region (Figs 3 and 5). Thus, replication of D4-275-184 containing targets for mir-275 and mir-184 in the 3’NCR was not detected in either Aag2 or C6/36 cells, whereas D4-E containing the same targets in the duplicated E-NS1 region replicated to titers of 2.5 and 5.5 log_10_ pfu/ml in these cells, respectively. Replication of D4-E in both cell lines was not due to loss of the miRNA targets and generation of escape mutants as was verified by sequence analyses (S3 Fig). In addition, combined expression of mir-275 and mir-184 targets in the 3’NCR was sufficient to completely block the D4-275-184 virus infectivity for *A*. *aegypti* and *A*. *albopictus* (Fig 4), whereas D4-E was detected in 5.6% (1/24) of *A*. *aegypti* mosquitoes (Fig 6). These findings are consistent with the notion that cellular mRNA translational repression occurs more frequently and efficiently by miRNA-targeting of mRNAs in the 3’NCR compared to that in the ORF or 5’NCR, presumably due to a lack of interference between the RISC and polysomes [45,46,60,61].

Surprisingly, infectivity of D4-275x2, containing two tandem targets for mir-275, in *A*. *aegypti* mosquitoes was significantly higher than that of single-copy targeted D4-275 virus (Fig 4). This phenomenon could be explained in part by assuming that introduction of a second mir-275 target results in the alteration in the 3’NCR secondary structure, which would make both mir-275 target sequences less accessible for miRNA-recognition and RISC binding. The m-fold RNA analysis predicts that two mir-275 targets could form a secondary structure with a 13 nt complementarity in the 3’NCR of the D4-275x2 genome (S4 Fig). The resulting structure could sequester both target sequences from interactions with RISC, as was previously shown for miRNA targets located in the regions of extensive secondary RNA structures [62]. Interestingly, this sequestration effect of the mir-275 tandem target was observed only for D4-275x2 infectivity in *A*. *aegypti* mosquitoes, but not in *A*. *albopictus* mosquitoes or in Aag2 or C6/36 cells (Figs 3 and 4). This suggests that structural folding of the 3’NCR of D4-275x2 genome as well as assembly, recognition, and binding with RISCs might vary between cell lines and mosquito species, which probably mirrors variation in specific cellular factors involved in these complex interactions.

The miRNA-based strategy of targeted host range restriction of flavivirus replication does not necessarily have to be confined to development of environmentally safe LAV against flaviviruses, but also could be applied for LAV against other arboviruses in *Togaviridae* and *Bunyaviridae* families. Moreover, similar to what was previously demonstrated for gain-of-function studies of influenza A virus [29,63–65], the miRNA based approach of host range restriction could potentially provide an additional level of biosafety for laboratories that use flaviviruses with altered pathogenicity or transmissibility characteristics. This should mitigate a risk for potential spread of novel pathogens in the nature after accidents/exposures in bio-containment laboratories.

Another significant observation of this study was the fact that targeting of the DEN4 genome with mosquito specific miRNA does not interfere with the capacity of CNS-enriched mir-124 miRNA to restrict replication of mir-124 targeted DEN4 viruses in the brain of newborn mice (Fig 7). This confirms the hypothesis that simultaneous targeting of RNA virus genome with sequences complementary to miRNAs overexpressed in different hosts can result in independent attenuation of virus replication in more than one host- or cell-type. This suggests that simultaneous multiple miRNA targeting can be applied to the construction of complex genetic systems with replication restricted to a very narrow range of target cells (organisms). This technique should find its use not only in LAV development but also in studies of virus pathogenesis using in vivo *disease models* [23,54].

### Summary

Here, for the first time we demonstrate that a combined targeting of the mosquito-borne flavivirus genome can silence viral replication in two evolutionally distant species: mosquitoes and mice. We believe that the miRNA co-targeting approach can be adapted to support the design of environmentally safe, live attenuated virus vaccines by restricting their ability to be introduced into nature during feeding of competent vectors on viremic vaccinees, thereby limiting the possibility of subsequent viral evolution and unpredictable consequences. Thus, miRNA-mediated silencing of virus replication in mice and mosquitoes can reinforce the safety of newly developed and existing vaccines for use in humans. This engineered host range restriction in both insect vector and vertebrate host by miRNA-mediated mechanisms represents an alternative to non-specific strategies for the rational control of viral tissue tropism and pathogenesis in the vertebrate host and replicative efficacy in permissive vectors.

## Materials and Methods

### Plasmids

Recombinant cDNA clone of dengue type 4 virus strain 814669, referred to here as D4s (GenBank access # AY648301) has been described previously [40]. To generate a modified D4 version of D4s clone, DNA fragments of viral genome were amplified from D4s using Phusion DNA polymerase (New England Biolabs [NEB], Ipswich, MA) and cloned individually or in combinations into low copy number pACNR1181 plasmid vector [66] using conventional PCR-based methods [67]. Intron sequence [nt. 857–989 in HaloTag CMV-neo vector pHTC (GenBank access # JF920305)] was synthesized by GenScript Inc. (Piscataway, NJ) and was introduced at positions 3922 and 8836 of DEN4 genome at AGCT sites. cDNA of CMV promoter was amplified from pCMV-SPORT6 plasmid (Invitrogen, Carlsbad, CA) and was fused with 5' end of DEN4 genome using PCR-based techniques. The hepatitis delta ribozyme sequence was amplified from plasmid RBZ-17D/25C-GFP-FMDV2A-YFCO_full_prME (generous gift of Dr. I. Frolov, University of Alabama), and fused with polyA signal/translation terminator sequence that was amplified from pTriEx1.1 (Novagen, Germany). The resulting amplicon was fused with the 3’-end of DEN4 genome to assemble wild-type D4 cDNA clone. A Vero cell adaptive mutation L_122_→F [41–44] was introduced into NS4B protein gene to generate a D4 plasmid.

Target sequences for mosquito specific mir-1 (5’-CTCCATACTTCTTTACATTCCA-3’), mir-184 (5’-GCCCTTATCAGTTCTCCGTCCA-3’) and mir-275 (5’-GCGCTACTTCAGGTACCTGA-3’) or human brain-specific mir-124 (5’-GGCATTCACCGCGTGCCTTA-3’) were introduced into the 3’NCR of DEN4 genome between nts 10,277 and 10,278 (position 1, Fig 1) or 10,474 and 10,475 (position 2, Fig 1); these sites of target insertion are located 15 or 212 nts downstream of the TAA stop codon in the 3’NCR, respectively. To introduce miRNA targets in the ORF, we utilized the approach that has been described previously [34,47]. Specifically, the introduced sequence was inserted between nts 2451 and 2452 of DEN4 genome and contained five tandem targets for mir-124, mir-184 and mir-275 that were followed by a duplicated DEN4 E/NS1 region (nts from 2130 to 2451 of DEN4 genome) encoding 98 amino acids from the C-terminal end of the DEN4 E protein and 7 amino acids from the N-terminal end of the NS1 protein (Fig 1). Each codon (except for Met and Trp) within the duplicated E/NS1 region was mutated to a synonymous codon to minimize nucleotide sequence homology between repeated DEN4 genome segments (Fig 1). Each plasmid DNA was sequenced to verify its integrity. Detailed information for all plasmids is available from the authors upon request.

### Cells and mosquitoes

Vero cells (African green monkey kidney) were cultured in serum free Opti-Pro medium (Invitrogen) as previously described [42]. Mosquito C6/36 (derived from *A*. *albopictus*; ATCC), C_7_10 and Aag2 (derived from *A*. *albopictus* and *A*. *aegypti*, respectively; generous gift from Dr. A. Fallon, University of Minnesota) cells were maintained in Dulbecco minimal essential medium (DMEM) (Invitrogen) supplemented with 5% FBS (Lonza), 1x penicillin-streptomycin-glutamine (PSG) solution (Invitrogen), 1x MEM nonessential amino acids (Cellgro, Swedesboro, NJ), 1 × MEM vitamin solution (Invitrogen) and 5 μg/L of gentamicin (Invitrogen) at 32°C in an atmosphere of 5% CO_2_. The C6/36 cells were used in all but the initial experiments, because they exhibit superior viability in our experimental conditions compared to C_7_10 cells.

Galveston colony of *A*. *albopictus* (generous gift of Dr. S. C. Weaver, UTMB) and NIH strain of *A*. *aegypti* mosquitoes have been described earlier [53,68]. Both mosquito species were maintained in carton containers supplemented with 10% sucrose on cotton balls at 28°C, 80% humidity and with a 16-hr daylight cycle.

### Virus recovery

#### 1) DNA transfection (D4 and its derivatives)

Twenty-four hours before transfection, 1.2x10^6^ low-passage Vero cells were seeded into a 12.5 cm^2^ flask in 5 mL of DMEM media supplemented with 10% FBS and 1x PSG solution. Next day, Vero cells were transfected with 5 μg of plasmid DNA using Lipofectamine 2000 reagent (Invitrogen) according to manufacturer’s instructions. Cells were maintained in DMEM at 37°C, 5% CO_2_ for 5 days. Cell culture supernatant was harvested, and virus was titrated on Vero cell monolayers and was purified by one-step terminal dilution as described earlier [42]. Experimental virus stocks were prepared by two consecutive passages in Vero cells (passages 143–149) maintained in Opti-Pro (Invitrogen), followed by titration of viruses in Vero cells. Complete genomes of biologically-cloned viruses were sequenced to ensure genetic integrity.

#### 2) RNA electroporation (D4s and its derivatives)

Five micrograms of plasmid DNA was linearized with *Age*I restriction endonuclease (NEB, Ipswich, MA), followed by *in vitro* RNA transcription from the SP6 promoter using the mMESSAGE mMACHINE kit (Ambion, Austin, TX) according to manufacturer's instructions. Ten micrograms of RNA was electroporated into 5x10^7^ Vero cells using a Gene Pulser Xcell electroporation system (Bio-Rad, Hercules, CA) with 4-mm cuvettes at the following conditions: 300V, pulse length 10μs, 5 pulses, with an interval between the pulses of 1s. Cells were transferred to a 75 cm^2^ flask and allowed to recover in 14 mL of Leibovitz’s L-15 medium (Invitrogen) supplemented with 10% FBS for 4 h at 37°C. The media was replaced with 14 mL of DMEM with 10% FBS, 1x PSG, and cells were incubated for 5 days at 37°C with 5% CO_2_, followed by harvest and titration in Vero cells. Experimental virus stocks were amplified by an additional passage in Vero cells maintained in Opti-Pro, and titrated on Vero cell monolayers. Presence of introduced miRNA targets in recovered viruses was verified by sequence analysis of viral cDNA genome.

To compare infectivity of *in vitro* transcribed RNAs, ten-fold dilutions of electroporated Vero cells were seeded in 1 mL of Opti-Pro media in 24-well plates containing 1x10^5^ cells. Cells were incubated for 4h at 37°C, and culture media was replaced with Opti-MEM (Invitrogen) containing 1% methylcellulose (Invitrogen) and 2% FBS. After incubation for 5 days at 37°C, the cells were fixed with 100% methanol, and plaques were visualized by immunostaining with 4G2 monoclonal antibodies as described previously [42,43]. Infectious foci were counted and normalized to the number of cells used for electroporation.

### Multicycle replication of miRNA targeted viruses in vertebrate and mosquito cells lines

To determine effect of miRNA targeting on DEN4 replication, miRNA targeted or control viruses were diluted in Opti-Pro medium supplemented with 2% FBS, 2 mM L-glutamine and were used to infect Vero, C6/36, Aag2 or C_7_10 cells at an MOI of 0.01 in duplicate wells of a 6-well plate for 1 h at 37°C (Vero) or 32°C (mosquito cells). The cells were washed three times with Opti-Pro and 2.75 mL of fresh media was added. Aliquots of 0.25 mL were harvested daily and stored at −80°C until virus titration. Differences in virus replication kinetics were compared using 2-way ANOVA analyses implemented in Prism 6 software (La Jolla, CA).

### Genetic stability of miRNA targeted viruses in simian Vero cells

Confluent monolayers of Vero cells in 25 cm^2^ flasks were infected at an MOI of 0.01 and cell culture supernatant was harvested at 3 dpi, diluted 1/10 with Opti-Pro medium, and 1 mL of inoculum was used to infect 25 cm^2^ flasks of fresh Vero cells. The cycle was repeated 5 times. At the end of the fifth passage, viral RNA was extracted from 0.14 mL of supernatant using the QIAamp Viral RNA Mini kit (Qiagen) according to manufacturer’s instructions. Genome regions flanking miRNA target sites were amplified using Titan One Tube RT-PCR kit (Roche, Indianapolis, IN) and sequenced.

### Evaluation of viruses in mosquitoes

#### 1) Intrathoracic infection (D4s and its derivatives)

Fifty 5-day-old female *A*. *aegypti* mosquitoes were immobilized on ice and inoculated intrathoracically with 1 μL of medium containing 5 log_10_ pfu/ml (100 pfu/mosquito) of DEN4 viruses. Mosquitoes were returned to carton containers, provided with 10% sucrose *ad libitum*, and held at 28°C and 80% humidity. After 7 days, live mosquitoes were collected with a hand aspirator, killed by chilling at -20°C for 5 min, and stored at -80°C.

#### 2) Oral infections (D4 and its derivatives)

To prepare infectious blood meals, viruses were diluted with Opti-Pro media supplemented with 2% FBS, 2 mM L-glutamine, and 1x SPG (218 mM sucrose, 6 mM L-glutamic acid, 3.8 mM KH_2_PO_4_, 7.2 mM K_2_HPO_4_, pH 7.2) to titers equal to least concentrated virus used in analysis. Virus aliquot of 1 mL was mixed with 1 mL of defibrinated rabbit blood (Spring Valley Laboratories, Woodline, MD), and blood meals were offered to 4–5 day old female mosquitos in a 37°C preheated water-jacketed feeder or using the Hemotek membrane feeding system (Discovery Workshops, UK) covered in stretched Parafilm (American National Can, Chicago, IL). Blood meal samples were collected for virus titration. Feeding proceeded for 45 min, followed by sorting of engorged mosquitoes (stage 3+ [69]) on ice. Engorged females were returned to cages and incubated at 28°C, 80% humidity and 16/8-hr day/night cycle for 14 days. Live mosquitos were collected and stored as described above.

Virus infection was analyzed by plaque-forming assay of each whole mosquito body (intrathoracic infection) or in each decapitated mosquito (oral infection). To assess virus dissemination in orally infected mosquitoes, head and body homogenates were prepared separately. Mosquito bodies and heads were triturated in 0.25 mL of L-15 medium supplemented with 1x SPG, 0.05 mg/mL of Ciprofloxacin, 0.06 mg/mL of Clindamycin and 0.0025 mg/mL of Amphotericin B, and homogenates were clarified by centrifugation at 10,000 rpm for 3 min. Head homogenate aliquots of 0.1 mL were used to infect Vero cells in one well of 24-well plates. Body homogenate samples were serially 10-fold diluted and 0.1 mL aliquots were used to infect monolayers of Vero cells in 24-well plates, followed by plaque visualization as described above. Mosquitoes were considered infected if at least one virus plaque was detected in the lowest dilution of body homogenate after 5 days incubation. Virus was considered to develop a disseminated infection if at least one virus plaque was detected in head homogenates after 5 days incubation. Differences in viral titers in mosquitos were compared using one-tailed Student's t-test, and P-values were adjusted using Bonferroni correction method to account for multiple comparisons (P = P’xN where N is number of comparisons). Differences in infection and dissemination frequencies were compared using one-tailed Fisher’s exact test and P-values were adjusted using Bonferroni correction method.

### Ethics statement

All animal experiments were done in compliance with the guidelines of the NIAID/NIH Institutional Animal Care and Use Committee. The NIAID DIR Animal Care and Use Program acknowledges and accepts responsibility for the care and use of animals involved in activities covered by the NIH IRP’s PHS Assurance #A4149-01, last issued 6/11/2011.

### Evaluation of viruses in sucking mice

D4 and miRNA targeted viruses were diluted to 5 log_10_ pfu/ml with L-15 medium supplemented with 1x SPG. Three-day-old Swiss Webster mice (Taconic Farms) in groups of 10 were inoculated intracranially (IC) with 10 μL (3 log_10_ pfu) of virus and returned to the dam. For study of virus replication, the brains from three mice were harvested on 5, 8, 11, and 14 days post-infection (dpi). Each brain was weighted, and 10% homogenates were prepared using L-15 medium supplemented with 1x SPG, 0.05mg/mL of Ciprofloxacin, 0.06 mg/mL of Clindamycin and 0.0025 mg/mL of Amphotericin B. Virus titers in each brain suspension were determined by titration in Vero cells. Harvesting of brains from mice infected with D4 was not performed at 14 dpi due to earlier death of the animals. To study the effect of miRNA targeting on virus neurovirulence, 3-day-old Swiss Webster mice (Taconic Farms) were inoculated IC with 3 log_10_ pfu of parental D4 or its miRNA-targeted derivative and monitored for morbidity and mortality for 21 dpi. Mice that developed neurological signs (paralysis) were humanely euthanized. Differences in replication kinetics were compared using 2-way ANOVA and P-values were adjusted using Bonferroni correction method to account for multiple comparisons, and differences in survival were compared using Log-rank (Mantel-Cox) test implemented in Prism 6 software (La Jolla, CA).

### Northern blot analysis of miRNA expression

The biotinylated probes complementary to mir-184 (5’Biotin-GCCCTTATCAGTTCTCCGTCCA-Biotin3’), mir-275 (5’Biotin-GCGCTACTTCAGGTACCTGA-Biotin3’), and mir-1 (5’Biotin-CTCCATACTTCTTTACATTCCA-Biotin3’) were synthesized by Bioresearch Technologies and used at 2–10 ng/mL. Ribo-oligonucleotides for artificial mir-184 (5’UGGACGGAGAACUGAUAAGGGC), mir-275 (5’UCAGGUACCUGAAGUAGCGC), and mir-1 (5’UGGAAUGUAAAGAAGUAUGGAG3’) were synthesized by Integrated DNA Technologies, and were used in northern blot as positive controls and molecular weight standards.

Total RNA was isolated with TRIzol SL reagent (Invitrogen) from 75-cm^2^ flasks containing confluent monolayers of Aag2, C6/36, C_7_10, or Vero cells or from individual brains of 5-day old Swiss mice or from pools of 20 adult *A*. *aegypti* mosquitoes collected at 7 days post-emergence. miRNA detection was carried out by northern blot as described previously with minor modifications [70]. A 15 μg sample of total RNAs was mixed with 4 μL of 10X RNA Loading Solution (Quality Biologicals) and then mixed with an equal volume of formamide (Sigma-Aldrich). The RNA was denatured at 90°C for 5 min followed by rapid cooling on ice. A total of 14 μg of denatured RNA was separated at 150 V for 1 h in a 15% polyacrylamide tris-borate-EDTA gel supplemented with 7M urea (BioRad). The gels were washed and stained with 100 mL of 20 mM MOPS-NaOH and 0.5 μg/mL ethidium bromide buffer (pH7.0) for 15 min. miRNAs was electroblotted to Amersham Hybond-NX membrane (GE Healthcare) at 20 V for 2 h in 10mM MOPS-NaOH buffer (pH7.0), followed by cross-linking to the membrane using 12 mL of 0.13 M 1-methylimidazole (Sigma), 0.16 M 1-ethyl-3-(3-dimethylaminopropyl) carbodiimide (Sigma) solution (pH 8.0) at 60°C for 1h. Membranes were prehybridized in 25 mL of ULTRAhyb ultrasensitive hybridization buffer (Ambion) at 37°C for 1 h, followed by hybridization with biotinylated probes at 39°C overnight in ULTRAhyb Ultrasensitive Hybridization Buffer. Membranes were then washed twice with 1x Low Stringency Washing Solution #1 (Ambion) and miRNAs were detected using Chemiluminescent Nucleic Acid Detection Module kit (Thermo scientific) according to the manufacturer's instructions.

## Supporting Information

S1 Fig Relative expression of mir-184 (A), mir-275 (B), mir-1 (C) in cell cultures, adult *A*. *aegypti* mosquitos, and new-born mouse brains. Total RNA was isolated from confluent monolayers of Aag2, C6/36, C_7_10, and Vero cells, from individual brains of 5-day old Swiss mice or from pools of 20 adult *A*. *aegypti* mosquitoes. For each line 14 μg of total RNA was used in northern blot analysis and then hybridized with biotinylated probes complementary to mir-184 (**A**), mir-275 (**B**), and mir-1 (**C**). As a loading control, the relative amount of 5.8S-5S rRNA/tRNA in each sample is shown in an ethidium bromide stained 15% polyacrylamide gel placed below each northern blot.
(TIF)

S2 Fig Genetic alteration of the 3’NCR of D4-184 and D4-275 viruses recovered at 14 dpi from bodies of *A*. *albopictus* and *A*. *aegypti* mosquitos. *A*. *albopictus* (**A**) and *A*. *aegypti* (**B**) *were* presented with blood meals containing 7.2 or 7.3 log_10_ pfu/mL of D4-184 or D4-275 virus, respectively. At 14 dpi DEN4-positive mosquitos were selected by titration of homogenized mosquito bodies in Vero cells as described in **Fig 4**. A 0.1 mL sample of homogenate from virus positive mosquito was used to infect one well of confluent monolayers of Vero cells in 24-well plates. At 5 days incubation, viral RNA was extracted, RT-PCR amplified, and the 3’NCR was sequenced. Each genomic representation corresponds to a virus isolated from one individual mosquito. Mir-184 and mir-275 target sequences are indicated as red and green boxes, respectively. Location of polyprotein stop codon (TAA), deleted nucleotides (brackets), size of deletion (Δnts), presence of point mutation in the target sequence (#) or mixed populations (^^^) are indicated.
(TIF)

S3 Fig Stability of miRNA targets in D4-E (A) and D4-E* (B) viruses in cell culture. Sequences of miRNA targets and duplicated DEN4 E/NS1 regions encoding 98 C-terminal amino acids of the DEN4 E protein and 7 N-terminal amino acids of the NS1 protein (nts. 2130 to 2451; “inserted sequence” in Fig 1) for D4-E (**A**) and D4-E* (**B**) are shown. Synonymous mutations introduced into mir-184 and mir-275 target sequences of D4-E* are highlighted in bold letters. Both viruses were passed repeatedly in Vero cells and sequence analysis was performed after the fifth passage. Viral RNA was extracted and the genome regions flanking the miRNA target sites were amplified and sequenced. Sequence electropherograms for genome regions containing miRNA targets in D4-E (**A**) and D4-E* (**B**) viruses are shown. Identical sequence data was obtained from D4-E virus after one passage in Aag2 and C6/36 cells.
(TIF)

S4 Fig Predicted secondary RNA structure of the 3’NCR of D4-275 and D4-275x2 viruses. The secondary RNA structures of D4-275 (**A**) and D4-275x2 (**B**) 3’NCRs were generated using MFOLD 3.2 accessed at http://mfold.rna.albany.edu/?q=mfold/RNA-Folding-Form using default parameters. The mir-275 targets sequences are indicated in lower case.
(TIF)

S1 Table Recovery of D4s derived viruses (electroporation into Vero cells). ^a^ Titers were determined 5 days post-electroporation. ^b^ Specific infectivity is expressed as the proportion of Vero cells yielding a productive viral infection after electroporation with 10 μg of RNA normalized to the total number of cells used for electroporation. ^c^ A 0.1 mL supernatant of transfected Vero cells was used to infect confluent monolayers of Vero cells. Viruses were harvested after 5 days and titrated on Vero cells. ^d^ Genetic stability of miRNA targets in working stock viruses was verified by sequence analysis of the 3’NCR (+).
(DOCX)

S2 Table Recovery of D4 derived viruses (Vero cell DNA transfection). ^a^ Titers were determined 5 days post-transfection. ^b^ Viruses were purified by one-step terminal dilution and experimental virus stocks were prepared by two consecutive passages in Vero cells, followed by titration of viruses in Vero cells. ^c^ Complete genomes of biologically cloned viruses were sequenced to ensure genetic integrity.
(DOCX)
