# Supplementary figures and images for: Dual miRNA Targeting Restricts Host Range and Attenuates Neurovirulence of Flaviviruses
Source: PLoS Pathog. 2015 Apr 23;11(4):e1004852. doi: 10.1371/journal.ppat.1004852 (PMC4408003; doi:10.1371/journal.ppat.1004852)

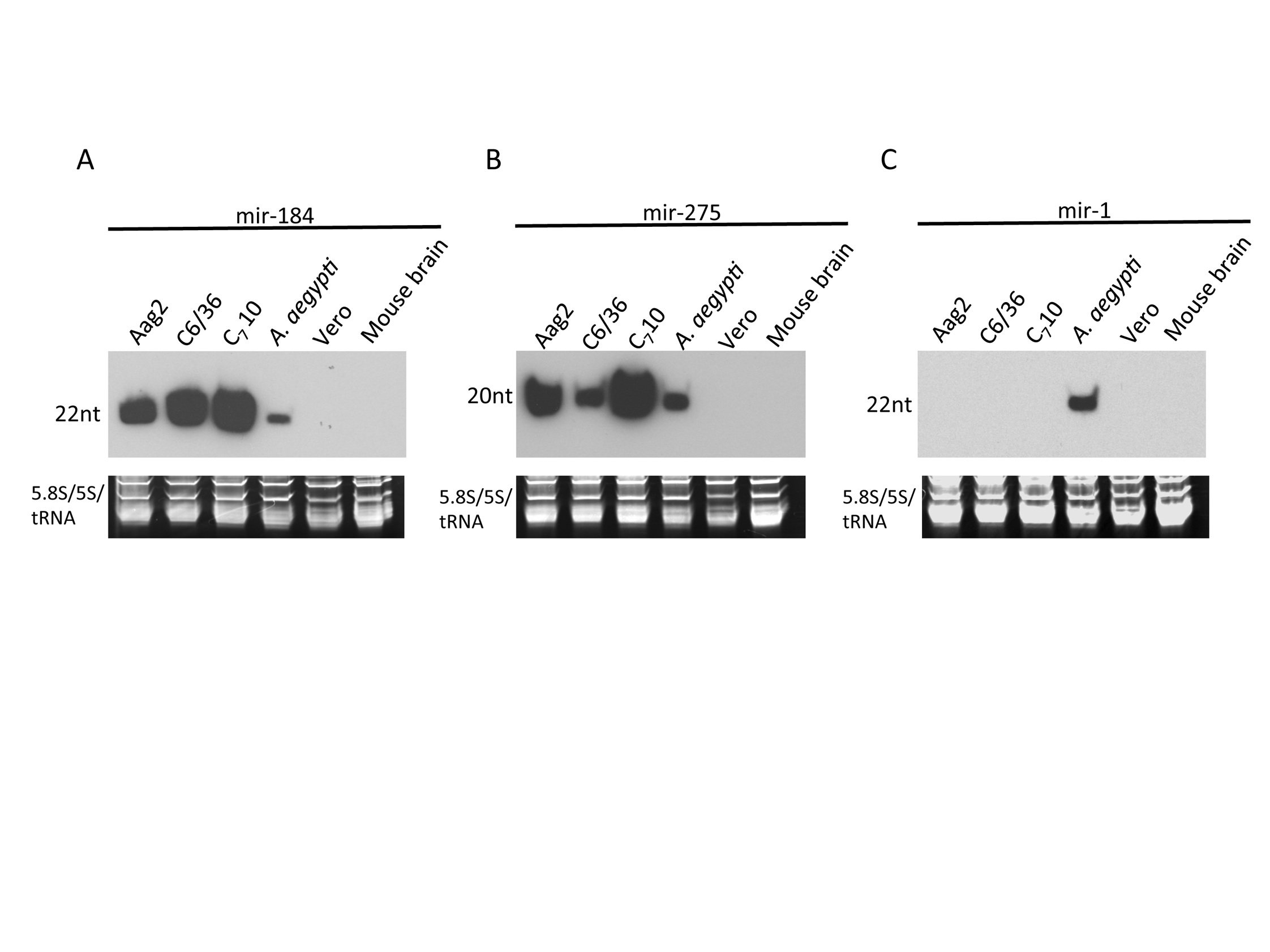

Supplement: S1 Fig — Total RNA was isolated from confluent monolayers of Aag2, C6/36, C710, and Vero cells, from individual brains of 5-day old Swiss mice or from pools of 20 adult A. aegypti mosquitoes. For each line 14 μg of total RNA was used in northern blot analysis and then hybridized with biotinylated probes complementary to mir-184 (A), mir-275 (B), and mir-1 (C). As a loading control, the relative amount of 5.8S-5S rRNA/tRNA in each sample is shown in an ethidium bromide stained 15% polyacrylamide gel placed below each northern blot. (TIF) [file ppat.1004852.s001.tif]

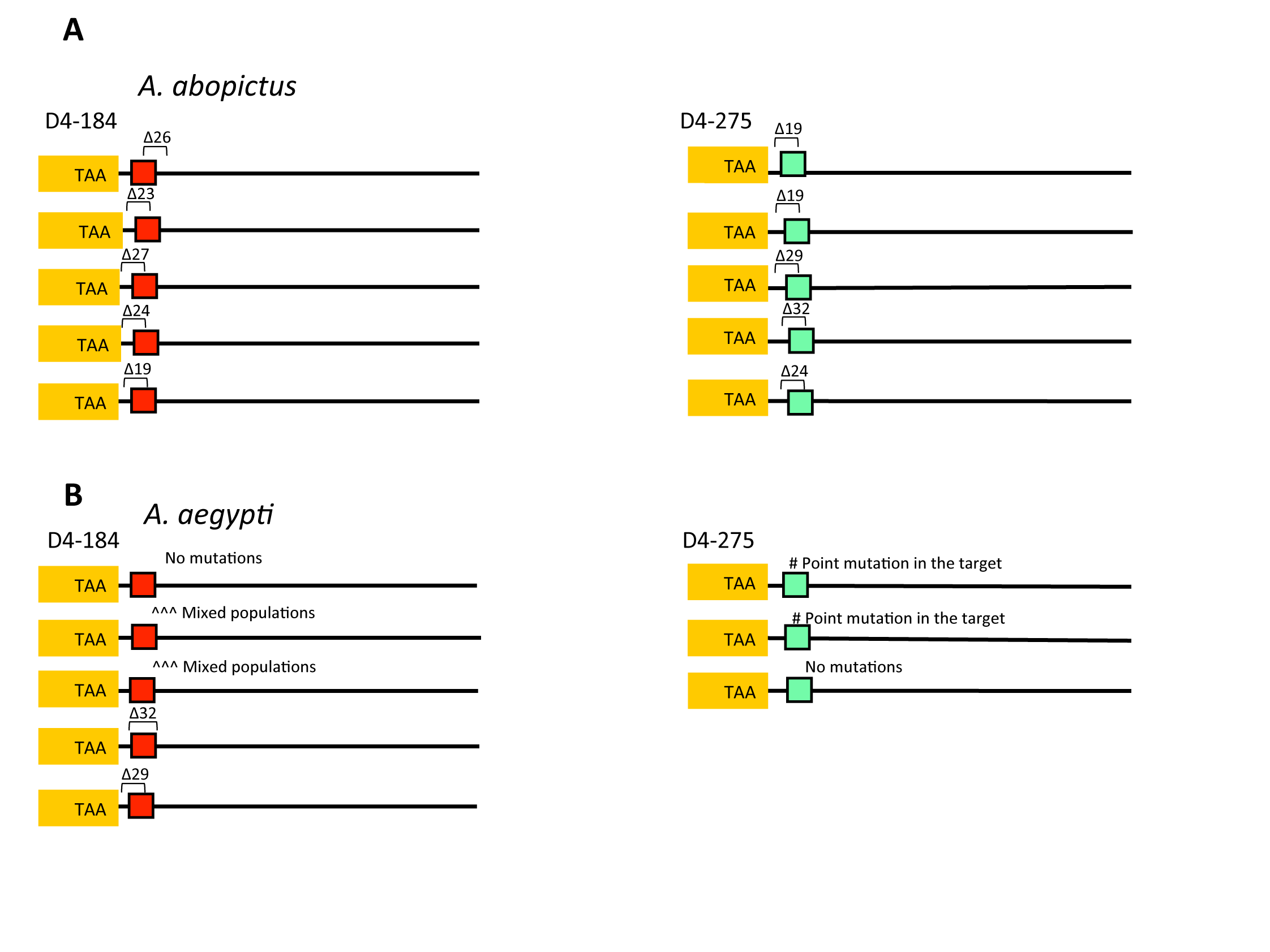

Supplement: S2 Fig — A. albopictus (A) and A. aegypti (B) were presented with blood meals containing 7.2 or 7.3 log10 pfu/mL of D4-184 or D4-275 virus, respectively. At 14 dpi DEN4-positive mosquitos were selected by titration of homogenized mosquito bodies in Vero cells as described in Fig 4. A 0.1 mL sample of homogenate from virus positive mosquito was used to infect one well of confluent monolayers of Vero cells in 24-well plates. At 5 days incubation, viral RNA was extracted, RT-PCR amplified, and the 3’NCR was sequenced. Each genomic representation corresponds to a virus isolated from one individual mosquito. Mir-184 and mir-275 target sequences are indicated as red and green boxes, respectively. Location of polyprotein stop codon (TAA), deleted nucleotides (brackets), size of deletion (Δnts), presence of point mutation in the target sequence (#) or mixed populations (^^^) are indicated. (TIF) [file ppat.1004852.s002.tif]

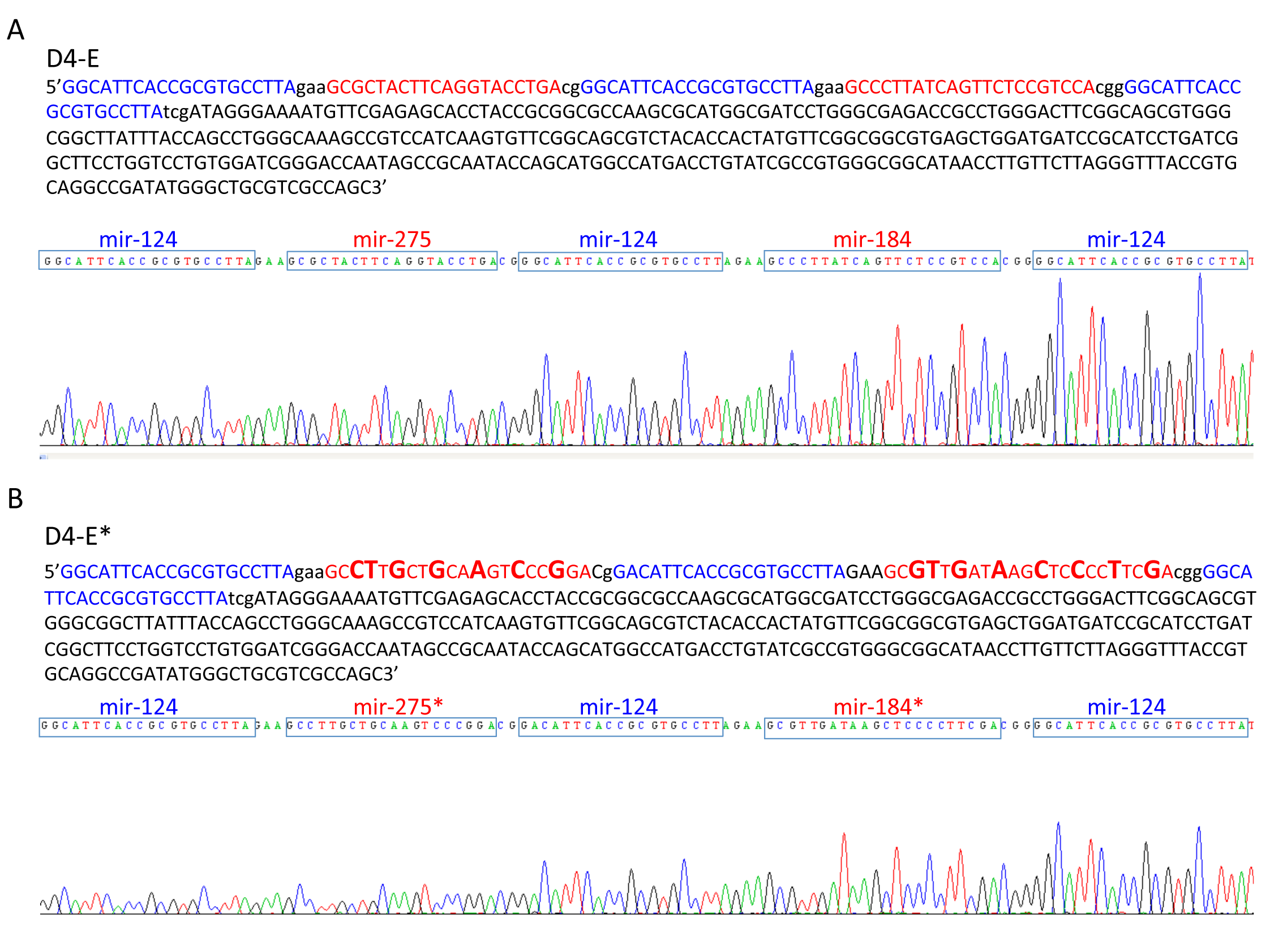

Supplement: S3 Fig — Sequences of miRNA targets and duplicated DEN4 E/NS1 regions encoding 98 C-terminal amino acids of the DEN4 E protein and 7 N-terminal amino acids of the NS1 protein (nts. 2130 to 2451; “inserted sequence” in Fig 1) for D4-E (A) and D4-E* (B) are shown. Synonymous mutations introduced into mir-184 and mir-275 target sequences of D4-E* are highlighted in bold letters. Both viruses were passed repeatedly in Vero cells and sequence analysis was performed after the fifth passage. Viral RNA was extracted and the genome regions flanking the miRNA target sites were amplified and sequenced. Sequence electropherograms for genome regions containing miRNA targets in D4-E (A) and D4-E* (B) viruses are shown. Identical sequence data was obtained from D4-E virus after one passage in Aag2 and C6/36 cells. (TIF) [file ppat.1004852.s003.tif]

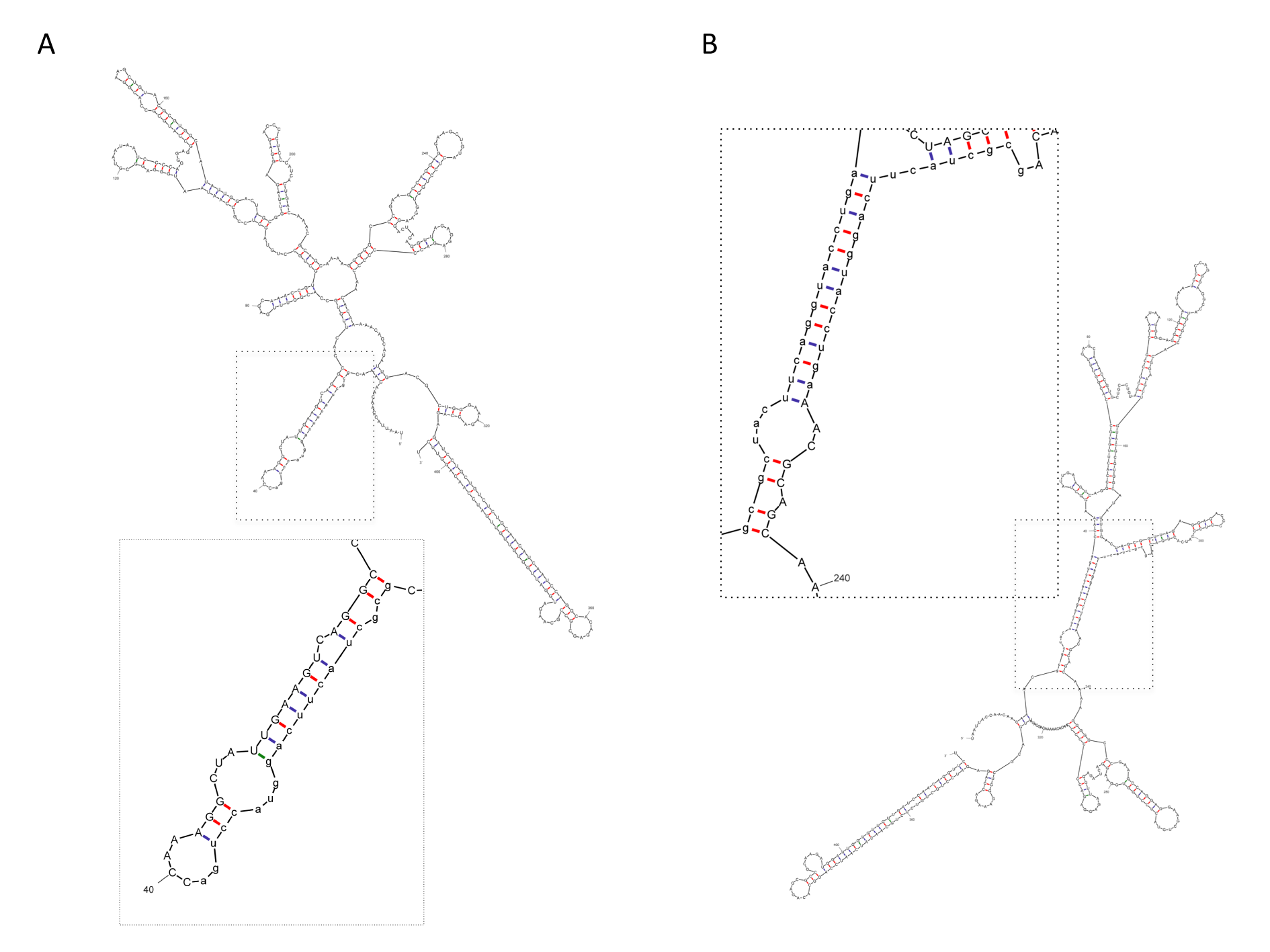

Supplement: S4 Fig — The secondary RNA structures of D4-275 (A) and D4-275x2 (B) 3’NCRs were generated using MFOLD 3.2 accessed at http://mfold.rna.albany.edu/?q=mfold/RNA-Folding-Form using default parameters. The mir-275 targets sequences are indicated in lower case. (TIF) [file ppat.1004852.s004.tif]
