# Supplementary material for: Dual miRNA Targeting Restricts Host Range and Attenuates Neurovirulence of Flaviviruses
Source: PLoS Pathog. 2015 Apr 23;11(4):e1004852. doi: 10.1371/journal.ppat.1004852 (PMC4408003; doi:10.1371/journal.ppat.1004852)
Supplement: S1 Table — a Titers were determined 5 days post-electroporation. b Specific infectivity is expressed as the proportion of Vero cells yielding a productive viral infection after electroporation with 10 μg of RNA normalized to the total number of cells used for electroporation. c A 0.1 mL supernatant of transfected Vero cells was used to infect confluent monolayers of Vero cells. Viruses were harvested after 5 days and titrated on Vero cells. d Genetic stability of miRNA targets in working stock viruses was verified by sequence analysis of the 3’NCR (+). (DOCX) [file ppat.1004852.s005.docx]

**S1 Table. Recovery of D4s derived viruses (electroporation into Vero cells)**

| Virus | miRNA target | Titer following transfection^a^  (log_10_pfu/mL) | Specific infectivity^b^ | Titer of working stock^c^  (log_10_pfu /mL) | Presence of miRNA targets^d^ |
| --- | --- | --- | --- | --- | --- |
| D4s | None | 4.7 | ∼5% | 7.1 | N/A |
| D4-184s | mir-184 | 3.5 | ∼5% | 6.3 | + |
| D4-275s | mir-275 | 5.0 | ∼5% | 6.6 | + |
| D4-1s | mir-1 | 5.2 | ∼5% | 6.9 | + |
| D4-275-184s | mir-275-184 | 1.0 | ∼0.05% | 4.4 | + |
| D4-275x2s | mir-275x2 | 1.3 | ∼0.05% | 5.2 | + |

^a^ Titers were determined 5 days post-electroporation.

^b^ Specific infectivity is expressed as the proportion of Vero cells yielding a productive viral infection after electroporation with 10 µg of RNA normalized to the total number of cells used for electroporation.

^c^ A 0.1 mL supernatant of transfected Vero cells was used to infect confluent monolayers of Vero cells. Viruses were harvested after 5 days and titrated on Vero cells.

^d^ Genetic stability of miRNA targets in working stock viruses was verified by sequence analysis of the 3’NCR (+).
