# Supplementary material for: Dual miRNA Targeting Restricts Host Range and Attenuates Neurovirulence of Flaviviruses
Source: PLoS Pathog. 2015 Apr 23;11(4):e1004852. doi: 10.1371/journal.ppat.1004852 (PMC4408003; doi:10.1371/journal.ppat.1004852)
Supplement: S2 Table — a Titers were determined 5 days post-transfection. b Viruses were purified by one-step terminal dilution and experimental virus stocks were prepared by two consecutive passages in Vero cells, followed by titration of viruses in Vero cells. c Complete genomes of biologically cloned viruses were sequenced to ensure genetic integrity. (DOCX) [file ppat.1004852.s006.docx]

**S2 Table. Recovery of D4 derived viruses (Vero cell DNA transfection)**

| Virus | Titer following transfection^a^  (log_10_pfu/mL) | Titer of working stock^b^  (log_10_pfu/mL) | Presence of miRNA targets^c^ |
| --- | --- | --- | --- |
| D4 (wt) | 7.4 | 7.9 | N/A |
| D4-184 | 7.1 | 7.7 | + |
| D4-275 | 7.2 | 7.7 | + |
| D4-275-184 | 6.4 | 7.4 | + |
| D4-275x2 | 6.6 | 7.4 | + |
| D4-E | 7.2 | 7.8 | + |
| D4-E^*^ | 7.2 | 7.8 | + |
| D4-E^**^ | 7.6 | 7.5 | + |
| D4-E-NCR1 | 6.2 | 7.5 | + |
| D4-E-NCR2 | 5.6 | 7.2 | + |

^a^ Titers were determined 5 days post-transfection.

^b^ Viruses were purified by one-step terminal dilution and experimental virus stocks were prepared by two consecutive passages in Vero cells, followed by titration of viruses in Vero cells.

^c^ Complete genomes of biologically cloned viruses were sequenced to ensure genetic integrity.

N/A - not applicable.
